# Food Intake and Diet Quality of Pregnant Women in China During the COVID-19 Pandemic: A National Cross-Sectional Study

**DOI:** 10.3389/fnut.2022.853565

**Published:** 2022-04-05

**Authors:** Haitian Chen, Hailin Li, Yinli Cao, Hongbo Qi, Yuyan Ma, Xiaoxia Bai, Yangyu Zhao, Li Wu, Caixia Liu, Jun Wei, Hong Wang, Yan Jin, Zilian Wang, Yanna Zhu

**Affiliations:** ^1^Department of Obstetrics and Gynecology of the First Affiliated Hospital, Sun Yat-sen University, Guangzhou, China; ^2^Department of Maternal and Child Health, School of Public Health, Sun Yat-sen University, Guangzhou, China; ^3^Department of Obstertrics, Northwest Women and Children Hospital, Xi'an, China; ^4^Department of Gynecology and Obstetrics, The First Affiliated Hospital of Chongqing Medical University, Chongqing, China; ^5^Department of Gynecology and Obstetrics, Qilu Hospital, Shandong University, Ji'nan, China; ^6^Department of Obstertrics, Women's Hospital, School of Medicine, Zhejiang University, Hangzhou, China; ^7^Department of Gynecology and Obstetrics, Peking University Third Hospital, Beijing, China; ^8^Reproductive Medical Center, Tongji Hospital, Tongji Medical College, Huazhong University of Science and Technology, Wuhan, China; ^9^Obstetrics and Gynecology at Shengjing Hospital of China Medical University, Shenyang, China; ^10^Nutrition Department, The International Peace Maternity and Child Health Hospital of Medicine College Shanghai Jiaotong University, Shanghai, China

**Keywords:** food intake, diet quality, variations, COVID-19 pandemic, severity, pregnancy

## Abstract

**Background:**

Between January and April 2020, China implemented differentiated prevention and control strategies across the country, based on the severity of the COVID-19 epidemic/pandemic in different regions. These strategies included lockdowns, social distancing, and the closure of public places. These measures may have affected dietary intake to varying degrees. This study aimed to assess variations in food intake and diet quality among pregnant women according to regional severity and related control measures during the most severe period of COVID-19 restrictions in 2020.

**Methods:**

A total of 3,678 pregnant women from 19 provinces/municipalities in mainland China were analyzed in this nationwide, multi-center study. Food intake data were obtained and assessed using a validated food frequency questionnaire (FFQ). Diet quality was quantified using the Diet Balance Index for Pregnancy (DBI-P), which included high bound score (HBS, excessive dietary intake), low bound score (LBS, insufficient dietary intake), and diet quality distance (DQD, dietary imbalance). Linear trend tests and multivariable regression analyses were performed to examine the association between food intake, DBI-P and the severity of pandemic.

**Results:**

The median daily intake of vegetables, fruit, livestock/poultry meat, dairy, and nuts decreased (*p* < 0.05) according to low, moderate, and high severity of the pandemic, while no significant differences in cereals/potatoes, eggs, and fish/shrimp intake. The median daily intake of cereals/potatoes exceeded the recommended ranges, and the daily intake of eggs and fish/shrimp was below recommended ranges regardless of the pandemic severity (*p* < 0.05). Regarding diet quality, HBS decreased (lower excessive consumption) (*p* = 0.047) and LBS increased (greater insufficient consumption) (*p* = 0.046) with increased severity of the pandemic. On multivariable analyses, moderate and high pandemic severity were related to lower HBS risk (OR = 0.687, OR = 0.537) and higher LBS risk (β = 1.517, β = 3.020) when compared to low pandemic severity.

**Conclusions:**

Under more severe COVID-19 pandemic conditions, pregnant women consumed less quality food, characterized by reduced consumption of vegetables, fruit, livestock/poultry meat, dairy and nuts, while the quality of the foods that pregnant women consumed in excess tended to improve, but the overconsumption of cereals/potatoes was a problem.

## Introduction

Due to the increase and distribution of newly reported cases of COVID-19 and the rapidly increasing death toll, on 11 March 2020, the World Health Organization (WHO) declared the coronavirus disease 2019 (COVID-19) a global pandemic (1). In early 2020, during the most severe period of COVID-19 restrictions, the Chinese government imposed strict sanitary and mitigation measures (2), causing the total confinement of the Chinese population until April 2020 (3). Under strict lockdown rules, the entire population was required to stay at home. Leaving the home was only permitted for the purposes of buying food and other essential supplies, thus fewer shopping opportunities and limiting social gatherings (4). Lockdown resulted in sudden and rapid changes to food purchasing behaviors and eating patterns. For example, people limited the frequency of food purchasing, purchased relatively large quantities of foods at one time, stocked up on food and no longer ate out in restaurants (5–8). Recent studies have reported changes to the dietary habits of the general population due to COVID-19 and related control measures (9–11). However, little or no attention has been paid to changes to the diets of pregnant women during this period of home confinement.

Pregnant women are potentially highly vulnerable to infectious diseases (12), and changes in the immune systems can affect their susceptibility to COVID-19 (13). Therefore, in the face of a public health emergency, such as COVID-19, pregnant women are likely to be more seriously affected than the general population. In addition, lifestyle changes, such as lack of physical activity, irregular sleep schedules, and the shopping inconvenience caused by severe pandemic control measures, may have caused pregnant women to be more severely affected by dietary change.

Between January and April 2020, Chinese authorities imposed full lockdown conditions in Wuhan (14), and carried out differentiated prevention and control measures in other regions, according to the differentiated severity of the pandemic. These control measures included the closure of public places, the cancellation or postponement of all public gatherings, isolation in the home, remote work, and online teaching (15). Food outlets were subject to differentiated control regimens according to regional rules. In regions where the pandemic situation was particularly severe, food markets and restaurants were closed on a large scale, while in areas where the pandemic was less severe, food markets and convenience stalls remained open under strict health and safety regulations. Under these circumstances, the food supply chain differed across the country, which may have had diverse and profound implications for the quantity and quality of food available in different areas.

Wuhan, the capital city of Hubei Province, urgently announced a lockdown from January 23, 2020. Under lockdown measures, pregnant women faced challenges to their antenatal care, medication use, and childbirth (16) due to transport restrictions and the strain on medical resources caused by the pandemic. Furthermore, faced with a surge of COVID-19 cases and hospitalizations, pregnant women faced the possibility of becoming infected. Because of these strains, pregnant women in Wuhan during this time were reported to have experienced higher than usual levels of depression and anxiety (17). As with other higher-risk groups elsewhere, pandemic-related stress may have led to changes in food intake, such as the consumption of more high energy foods (18, 19). Unhealthy eating patterns have been linked to increased risk of inflammatory conditions and respiratory disease (20, 21). To protect the future safety of pregnant women and promote their health in the post-pandemic period, the quantity and quality of food consumed by pregnant women in Hubei Province is of particular concern, given the pandemic severity in that province.

To illustrate these issues, this study sought to evaluate the intake and quality of foods consumed by pregnant women, as well as regional variations in consumption according to the severity of the COVID-19 pandemic and related control measures. This study also examined associations between diet quality and pandemic severity, demographic factors, and health-related behaviors among pregnant women.

## Materials and Methods

### Study Design and Participants

This nationwide, multi-center, cross-sectional survey was conducted from March 5 to April 16, 2020, with a sample of 3689 pregnant women from 19 provinces/municipalities distributed across seven geographical regions (Northeast China, North China, Central China, East China, South China, Northwest China, and Southwest China) representing the country's vast and varied geography, economy, and governance.

**Figure 1:**
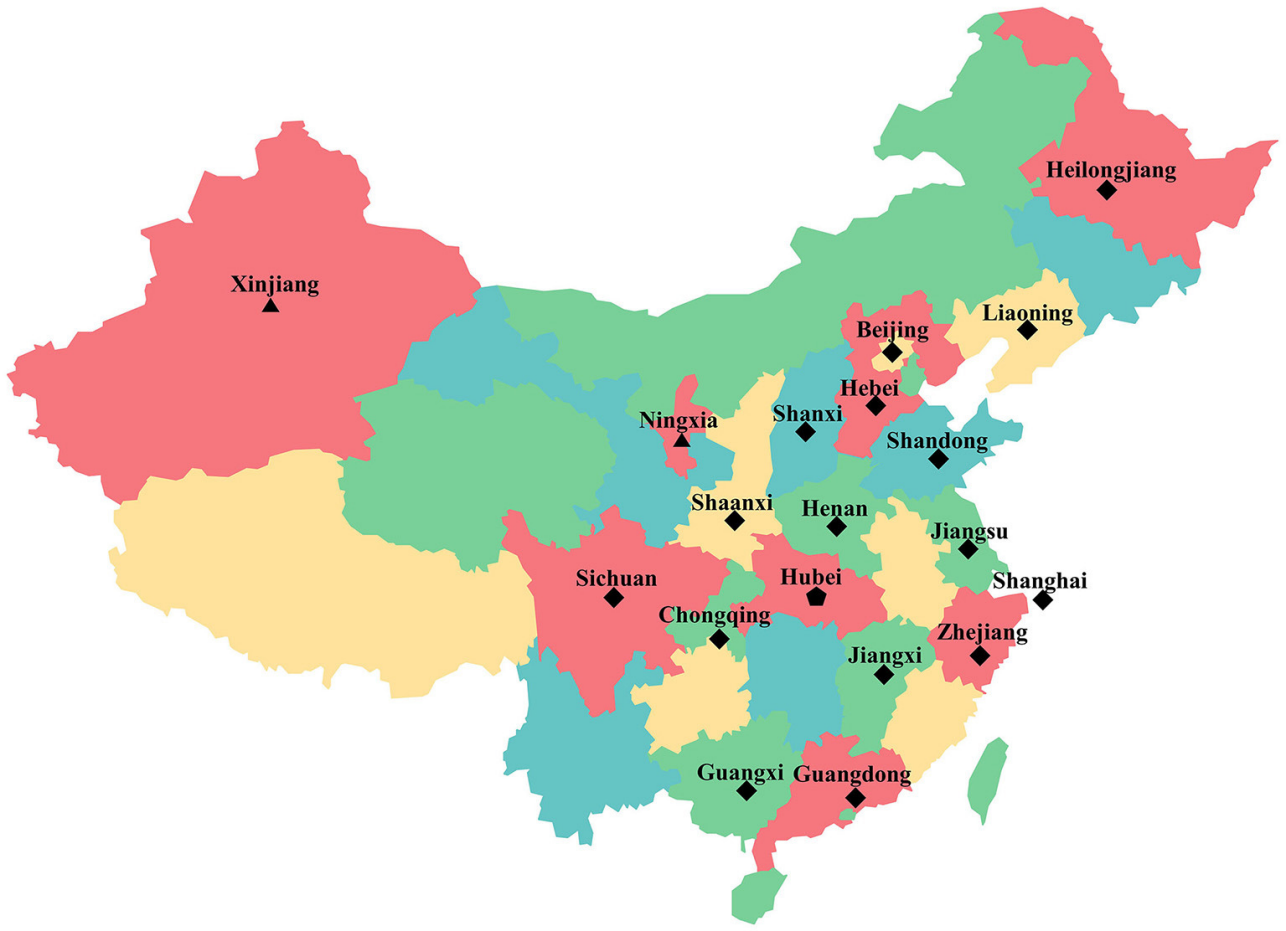
Geographic distribution of samples provinces/municipalities. The symbol represents the provinces/municipalities of different COVID-19 pandemic severity. 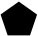
represents the pandemic severity was high. 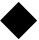
represents the pandemic severity was moderate. 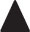 represents the pandemic severity was low.

A multistage sampling method was used to enroll study participants. Two to four provinces/municipalities were selected from each of the seven geographical regions, except for Hubei Province, which was the only representative of Central China, and 19 provinces/municipalities were eventually incorporated into the study (Figure 1). Finally, one hospital sample was randomly selected from each of the 19 provinces/municipalities. Pregnant women were recruited using online and offline methods at these selected hospitals. A total of 3,689 pregnant women from 19 hospitals in 19 provinces/municipalities across China were enrolled in the study. The study was conducted in accordance with the guidelines of the Declaration of Helsinki and underwent ethical review and approval by the Ethics Committee of the School of Public Health, Sun Yat-Sen University (No. 017 [2020]). All participants provided signed informed consent.

This study used data collected via an online questionnaire about participants' demographics, food intake frequency, and health-related behaviors between January 23 and February 29, 2020 (the “emergency phase” of the COVID-19 epidemic prevention and control). Of the original sample, a total of 3,678 pregnant women were included in the final statistical analysis following the removal of participants reporting implausible food intake (valid questionnaire rate: 99.7%).

### Assessment of Food Intake and Diet Quality

Using a food frequency questionnaire (FFQ), participants recalled and reported their usual frequency of food consumption between January 23 and February 29, 2020 as the number of times per day, per week, per month, or never, and the average quantity of food consumed each time they ate, to finally obtain the number of grams of food per day. The FFQ used in this study, designed specifically for urban Chinese pregnant women, had been evaluated and validated by Yang et al. The FFQ exhibited acceptable reproducibility and reasonable validity in assessing food intakes among pregnant women (22). The FFQ includes eight food groups, including cereals/potatoes, vegetables, fruit, livestock/poultry meat, fish/shrimp, eggs, nuts, and dairy, all of which are recommended in the Dietary Guidelines for Chinese Residents (2016) (23).

To capture adherence to dietary recommendations during the COVID-19 pandemic, diet quality was quantified using the Diet Balance Index for Pregnancy (DBI-P), established with reference to the DBI_16 (for Chinese adults) (24) constitutive indicators, scoring method, evaluation index, and criteria.

The DBI-P score measured intake of the eight aforementioned food groups and dietary variety. This comprised rice and its products, flour and its products, coarse grain/potatoes, vegetables, fruit, livestock meat, poultry meat, fish/shrimp, eggs, nuts, and dairy. According to dietary guidelines, cereals/potatoes, livestock/poultry meat, and eggs should be consumed in moderation, and a negative, zero, or positive score was computed to assess the degree of insufficient or excessive intake. Sufficient vegetables, fruit, fish/shrimp, nuts, and dairy should be consumed, and a negative or zero score was computed to assess the degree of insufficient intake (see detailed scoring methods in Supplementary Table 2), and the specific cutoff values for the eight food groups were derived from the Chinese Balanced Dietary Pagoda for pregnant women (Supplementary Table 1).

The DBI-P has three main indicators of diet quality, including high bound score (HBS), low bound score (LBS), and diet quality distance (DQD). HBS is equal to the sum of all positive values, reflecting the degree of excessive consumption, ranging from 0–20 (0: no excessive consumption; 1–4: almost no excessive consumption; 5–8: low level excessive consumption; 9–12: moderate level excessive consumption; 13–20: high level excessive consumption). LBS is the sum of absolute values of negative scores, reflecting the degree of insufficient consumption, ranging from 0–56 (0: sufficient consumption; 1–8: almost sufficient consumption; 9–20: low level insufficient consumption; 21–32: moderate level insufficient consumption; 33–56: high level insufficient consumption). DQD is the sum of HBS and LBS, reflecting the degree of consumption imbalance, ranging from 0–56 (0: balanced consumption; 1–8: almost balanced consumption; 9–20: low level imbalanced consumption; 21–32: moderate level imbalanced consumption; 33–56: high level imbalanced consumption). In addition, energy-adjusted intakes of foods were calculated based on the residual method and then energy-adjusted DBI-P was obtained.

### Classification of COVID-19 Pandemic Severity

COVID-19 pandemic severity was graded according to the cumulative number of confirmed cases between January 23 and February 29, 2020 reported on the official website of the health commission of each province/municipality. Of 31 provinces/municipalities across China, Hubei Province had 66,907 laboratory-confirmed and clinically diagnosed cases, and its pandemic severity was defined as high (total cases >10,000). A cumulative total of 100 to 10,000 laboratory confirmed cases were reported in 23 provinces/municipalities, where the pandemic severity was defined as moderate. A total of <100 laboratory confirmed cases were reported in seven regions, where the pandemic severity was defined as low. Figure 1 highlights the 19 provinces/municipalities included in this study of different COVID-19 pandemic severity.

### Measurement of Demographic Characteristics and Health-Related Behaviors

Data collection included demographic variables and health-related behaviors between January 23 and February 29, 2020. Demographic characteristics included age, region, pre-pregnancy body mass index (BMI), levels of educational attainment, monthly income, assisted reproductive technology use, gestational anemia, gestational hyperthyroidism, gestational diabetes mellitus (GDM), and physical activity. The region was also divided into South and North by the Qinling Mountains and Huaihe River. Pre-pregnancy BMI was categorized according to the Chinese standard reference (25). Physical activity was calculated as multiplying the metabolic equivalent of physical activity (26) by the time spent engaged in physical activity per week (MET-hours/week), and categorized according to 25th, 50th, and 75th percentiles. Health-related behaviors comprised number of hospital visits, number of pathways to acquiring self-care and parenting information, frequency of use of household fetal heart monitor, and frequency of intake of nutritional supplements (folic acid and calcium). Table 1 presents details for the classification of demographic variables and health-related behaviors.

**Table 1:** Demographic characteristics of pregnant women.

	**First trimester**	**Second trimester**	**Third trimester**	**Total population**
	**(*n* = 391)**	**(*n* = 1,078)**	**(*n* = 2,209)**	**(*n* = 3,678)**
**COVID-19 pandemic severity**				
Low-severity	15 (3.8)	65 (6.0)	78 (3.5)	158 (4.3)
Moderate-severity	368 (94.1)	963 (89.3)	2,094 (94.8)	3,425 (93.1)
High-severity	8 (2.0)	50 (4.6)	37 (1.7)	95 (2.6)
**Age (years)**				
<25	32 (8.2)	88 (8.2)	130 (5.9)	250 (6.8)
25–30	214 (54.7)	538 (49.9)	1,067 (48.3)	1,819 (49.5)
>30	145 (37.1)	452 (41.9)	1,012 (45.8)	1,609 (43.7)
**Region**				
North	192 (49.1)	493 (45.7)	1,456 (65.9)	2,141 (58.2)
South	199 (50.9)	585 (54.3)	753 (34.1)	1,537 (41.8)
**Pre-pregnancy BMI (kg/m** ^**2**^ **)**				
Underweight <18.5	56 (14.3)	155 (14.4)	323 (14.6)	534 (14.5)
Normal weight 18.5–23.9	274 (70.1)	702 (65.1)	1,509 (68.3)	2,485 (67.6)
Overweight 24.0–27.9	43 (11.0)	159 (14.7)	297 (13.4)	499 (13.6)
Obese ≥28.0	18 (4.6)	62 (5.8)	80 (3.6)	160 (4.4)
**Educational level**				
Less than senior school	32 (8.2)	99 (9.2)	123 (5.6)	254 (6.9)
Senior school or technical secondary school	37 (9.5)	162 (15.0)	232 (10.5)	431 (11.7)
College degree or above	322 (82.4)	817 (75.8)	1,854 (83.9)	2,993 (81.4)
**Monthly income (RMB)**				
<5,000	221 (56.5)	635 (58.9)	1,144 (51.8)	2,000 (54.4)
5,000–10,000	140 (35.8)	341 (31.6)	829 (37.5)	1,310 (35.6)
>10,000	30 (7.7)	102 (9.5)	236 (10.7)	368 (10.0)
**Assisted reproductive technology**				
No	341 (87.2)	934 (86.6)	1,989 (90.0)	3,264 (88.7)
Yes	50 (12.8)	144 (13.4)	220 (10.0)	414 (11.3)
**Gestational complications**				
**Anemia**				
No	312 (79.8)	797 (73.9)	1,486 (67.3)	2,595 (70.6)
Yes	25 (6.4)	170 (15.8)	626 (28.3)	821 (22.3)
Not knowing	54 (13.8)	111 (10.3)	97 (4.4)	262 (7.1)
**Hyperthyroidism**				
No	339 (86.7)	967 (89.7)	2,092 (94.7)	3,398 (92.4)
Yes	7 (1.8)	30 (2.8)	48 (2.2)	85 (2.3)
Not knowing	45 (11.5)	81 (7.5)	69 (3.1)	195 (5.3)
**Diabetes mellitus**				
No	320 (81.8)	846 (78.5)	1,743 (78.9)	2,909 (79.1)
Yes	8 (2.0)	57 (5.3)	398 (18.0)	463 (12.6)
Not knowing	63 (16.1)	175 (16.2)	68 (3.1)	306 (8.3)
Physical activity (MET-hours/week)^*^				
≤ 56	114 (29.2)	269 (25.0)	552 (25.0)	935 (25.4)
57–86	79 (20.2)	259 (24.0)	588 (26.6)	926 (25.2)
87–130	95 (24.3)	261 (24.2)	559 (25.3)	915 (24.9)
≥131	103 (26.3)	289 (26.8)	510 (23.1)	902 (24.5)
The number of visits to hospital^*^				
0	106 (27.1)	170 (15.8)	265 (12.0)	541 (14.7)
1–2	197 (50.4)	663 (61.5)	1,172 (53.1)	2,032 (55.2)
≥3	88 (22.5)	245 (22.7)	772 (34.9)	1,105 (30.0)
The number of ways to acquire self-care and parenting knowledge^*^				
1–2	9 (2.3)	7 (0.6)	13 (0.6)	29 (0.8)
3	264 (67.5)	689 (63.9)	1,390 (62.9)	2,343 (63.7)
>3	118 (30.2)	382 (35.4)	806 (36.5)	1,306 (35.5)
Frequency of use of household fetal heart monitor^*^				
None	186 (47.6)	774 (71.8)	1,555 (70.4)	2,515 (68.4)
≥1 times per day	7 (1.8)	141 (13.1)	212 (9.6)	360 (9.8)
4–5 times per week	35 (9.0)	39 (3.6)	62 (2.8)	136 (3.7)
2–3 times per week	8 (2.0)	67 (6.2)	190 (8.6)	265 (7.2)
1–2 times per month	155 (39.6)	57 (5.3)	190 (8.6)	402 (10.9)
Frequency of intake of nutritional supplements^*^				
**Folic acid**				
None	124 (31.7)	583 (54.1)	1,380 (62.5)	2,087 (56.7)
1 time per day	229 (58.6)	396 (36.7)	608 (27.5)	1,233 (33.5)
4–5 times per week	19 (4.9)	41 (3.8)	66 (3.0)	126 (3.4)
2–3 times per week	9 (2.3)	28 (2.6)	64 (2.9)	101 (2.7)
1–2 times per month	10 (2.6)	30 (2.8)	91 (4.1)	131 (3.6)
**Calcium**				
None	293 (74.9)	449 (41.7)	501 (22.7)	1,243 (33.8)
1 time per day	64 (16.4)	509 (47.2)	1,363 (61.7)	1,936 (52.6)
4–5 times per week	12 (3.1)	53 (4.9)	144 (6.5)	209 (5.7)
2–3 times per week	7 (1.8)	41 (3.8)	146 (6.6)	194 (5.3)
1–2 times per month	15 (3.8)	26 (2.4)	55 (2.5)	96 (2.6)

*BMI, body mass index; RMB, Renminbi; MET, metabolic equivalent; COVID-19, coronavirus disease 2019*.

* *During the COVID-19 pandemic (between Jan 23 and Feb 29, 2020)*.

*The data are presented as frequency (percentages). Percentages may not total 100 because of rounding*.

### Statistical Analysis

Skewed food intake and HBS are expressed as the median (interquartile range, IQR) and compared via Mann-Whitney *U*-test and Kruskal-Wallis rank sum test. Normally distributed variables (LBS and DQD) are expressed as the mean (standard deviation, SD) and analyzed using one-way ANOVA and the least significant difference (LSD) test. Categorical variables are displayed as frequencies or percentages. Linear trend tests were applied to assess variations in food intake, HBS, LBS, and DQD according to COVID-19 pandemic severity. Multivariate ordinal logistic regression and linear regression were used to further examine associations between DBI-P and COVID-19 pandemic severity, demographic factors, and health-related behaviors. HBS as a dependent variable was categorized into three grades as no excessive consumption and almost no excessive consumption (control): 0–4; low and moderate level excessive consumption: 5–12; high level excessive consumption: 13–20. Stepwise backward elimination was applied to eliminate variables in the multivariate linear regression analyses.

All statistical analyses were conducted using SPSS software and drawings were created with GraphPad Prism 9. A two-tailed *P*-value ≤ 0.05 was considered statistically significant.

## Results

### Study Participants

Of the 3,678 surveyed women, 391 (10.6%) were in the first trimester of pregnancy, 1078 (29.3%) in the second, and 2209 (60.1%) in the third. Table 1 shows the demographic characteristics and health-related behaviors for each of the three trimesters.

Distribution of food intake and diet quality in the three trimesters during the COVID-19 pandemic are shown in Supplementary Table 3, and average daily food intake level and DBI-P are exhibited in Supplementary Figure 1.

### Food Intake According to COVID-19 Pandemic Severity

The significant differences were found in the percentages of pregnant women with vegetables and livestock/poultry meat consumption according to the regional severity of the COVID-19 pandemic. The percentages of pregnant women who had inadequate consumption of vegetables were 69.0, 62.2, and 81.1% under low, moderate and high pandemic severity (*p* < 0.001), respectively. The percentages of pregnant women who had inadequate consumption of livestock/poultry meat were 45.6, 51.7, and 65.3%, respectively; and the percentages of women who had excessive consumption of livestock/poultry meat were 39.2, 33.9, and 20.0% (*p* = 0.023), respectively (Table 2).

**Table 2:** Distribution of food intake and diet quality (DBI) for pregnant women according to COVID-19 pandemic severity.

	**Low-severity**	***P*-value†**	**Moderate-severity**	***P*-value^**§**^**	**High-severity**	***P*-value^**‡**^**	**Total population**	*P*-value*
	**(158)**		**(*n* = 3,425)**		**(*n* = 95)**		**(*n* = 3,678)**	
Cereals/potatoes		0.574		0.069		0.397		0.176
Inadequate	32 (20.3)		816 (23.8)		13 (13.7)		861 (23.4)	
Appropriate	14 (8.9)		275 (8.0)		8 (8.4)		297 (8.1)	
Excessive	112 (70.9)		2,334 (68.1)		74 (77.9)		2,520 (68.5)	
Vegetables		0.083		<0.001		0.035		<0.001
Inadequate	109 (69.0)		2,129 (62.2)		77 (81.1)		2,315 (62.9)	
Appropriate	49 (31.0)		1,296 (37.8)		18 (18.9)		1,363 (37.1)	
Fruit intake		0.224		0.927		0.400		0.474
Inadequate	63 (39.9)		1,534 (44.8)		43 (45.3)		1,640 (44.6)	
Appropriate	95 (60.1)		1,891 (55.2)		52 (54.7)		2,038 (55.4)	
Livestock/poultry meat		0.294		0.014		0.004		0.023
Inadequate	72 (45.6)		1,771 (51.7)		62 (65.3)		1,905 (51.8)	
Appropriate	24 (15.2)		494 (14.4)		14 (14.7)		532 (14.5)	
Excessive	62 (39.2)		1,160 (33.9)		19 (20.0)		1,241 (33.7)	
Fish/shrimp		0.789		0.084		0.175		0.220
Inadequate	142 (89.9)		3,055 (89.2)		90 (94.7)		3,287 (89.4)	
Appropriate	16 (10.1)		370 (10.8)		5 (5.3)		391 (10.6)	
Eggs		0.293		0.770		0.802		0.572
Inadequate	96 (60.8)		2,287 (66.8)		61 (64.2)		2,444 (66.4)	
Appropriate	16 (10.1)		293 (8.6)		10 (10.5)		319 (8.7)	
Excessive	46 (29.1)		845 (24.7)		24 (25.3)		915 (24.9)	
Nuts		0.913		0.151		0.221		0.352
Inadequate	84 (53.2)		1,836 (53.6)		58 (61.1)		1,978 (53.8)	
Appropriate	74 (46.8)		1,589 (46.4)		37 (38.9)		1,700 (46.2)	
Dairy		0.911		0.692		0.800		0.920
Inadequate	138 (87.3)		2,981 (87.0)		84 (88.4)		3,203 (87.1)	
Appropriate	20 (12.7)		444 (13.0)		11 (11.6)		475 (12.9)	
Dietary variety score		0.150		0.370		0.094		0.204
0	16 (10.1)		234 (6.8)		3 (3.2)		253 (6.9)	
−1 to −5	128 (81.0)		2,771 (80.9)		80 (84.2)		2,979 (81.0)	
−6 to −10	14 (8.9)		420 (12.3)		12 (12.6)		446 (12.1)	
HBS		0.482		0.088		0.076		0.166
No problem	30 (19.0)		681 (19.9)		16 (16.8)		727 (19.8)	
Almost no problem	25 (15.8)		708 (20.7)		24 (25.3)		757 (20.6)	
Low level problem	27 (17.1)		522 (15.2)		22 (23.2)		571 (15.5)	
Moderate level problem	39 (24.7)		854 (24.9)		22 (23.2)		915 (24.9)	
High level problem	37 (23.4)		660 (19.3)		11 (11.6)		708 (19.2)	
LBS		0.477		0.788		0.552		0.725
No problem	1 (0.6)		14 (0.4)		NA		15 (0.4)	
Almost no problem	32 (20.3)		560 (16.4)		12 (12.6)		604 (16.4)	
Low level problem	86 (54.4)		1,979 (57.8)		57 (60.0)		2,122 (57.7)	
Moderate level problem	33 (20.9)		680 (19.9)		22 (23.2)		735 (20.0)	
High level problem	6 (3.8)		192 (5.6)		4 (4.2)		202 (5.5)	
DQD		0.635		0.849		0.562		0.861
Almost no problem	2 (1.3)		30 (0.9)		NA		32 (0.9)	
Low level problem	56 (35.4)		1,198 (35.0)		36 (37.9)		1,290 (35.1)	
Moderate level problem	86 (54.4)		1,801 (52.6)		47 (49.5)		1,934 (52.6)	
High level problem	14 (8.9)		396 (11.6)		12 (12.6)		422 (11.5)	

*DBI, diet balance index; COVID-19, coronavirus disease 2019; HBS, high bound score; LBS, low bound score; DQD, diet quality distances; NA, not applicable*.

Statistically significant difference between ^†^First vs. second trimester; ^§^Second vs. third trimester; ^‡^First vs. third trimester; ^*^First vs. second vs. third trimester (p < 0.05). The data are presented as frequency (percentages). Percentages may not total 100 because of rounding.

**Figure 2:**
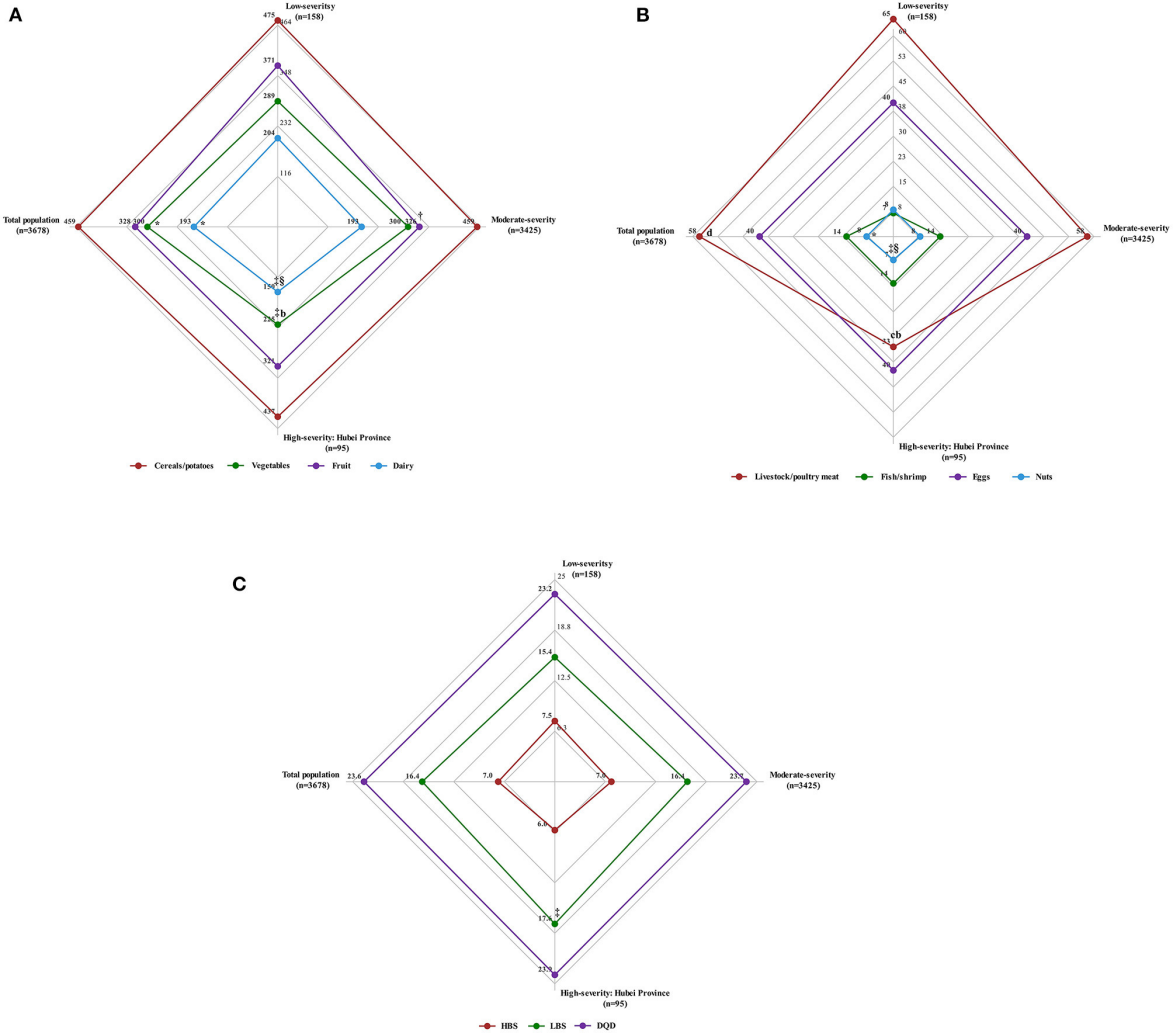
Average daily food intake and diet quality (Diet Balance Index, DBI) for pregnant women according to COVID-19 pandemic severity. **(A)** The median daily intake of cereals/potatoes, vegetables, fruit and dairy (g/day) for pregnant women according to COVID-19 pandemic severity. **(B)** The median daily intake of livestock/poultry meat, fish/shrimp, eggs and nuts (g/day) for pregnant women according to COVID-19 pandemic severity. **(C)** The median of high bound score (HBS), the mean of low bound score (LBS) and diet quality distances (DQD) for pregnant women according to COVID-19 pandemic severity. ^†^*p* < 0.05 (low vs. moderate severity). ^§^*p* < 0.05, ^b^*p* < 0.001 (moderate vs. high severity). ^‡^*p* < 0.05, ^c^*p* < 0.001 (low vs. high severity). *p < 0.05, ^d^*p* < 0.001 (low vs. moderate vs. high severity).

Figure 2 shows the median daily intake of various food groups in regions of low, moderate, and high pandemic severity. Notably, the average daily intakes of vegetables, livestock/poultry meat, dairy, and nuts were significantly lower in high severity than in moderate and low severity regions (*p* < 0.05). The average daily intake of fruit was significantly lower in moderately severe regions than in low severity regions (*p* = 0.023).

**Figure 3:**
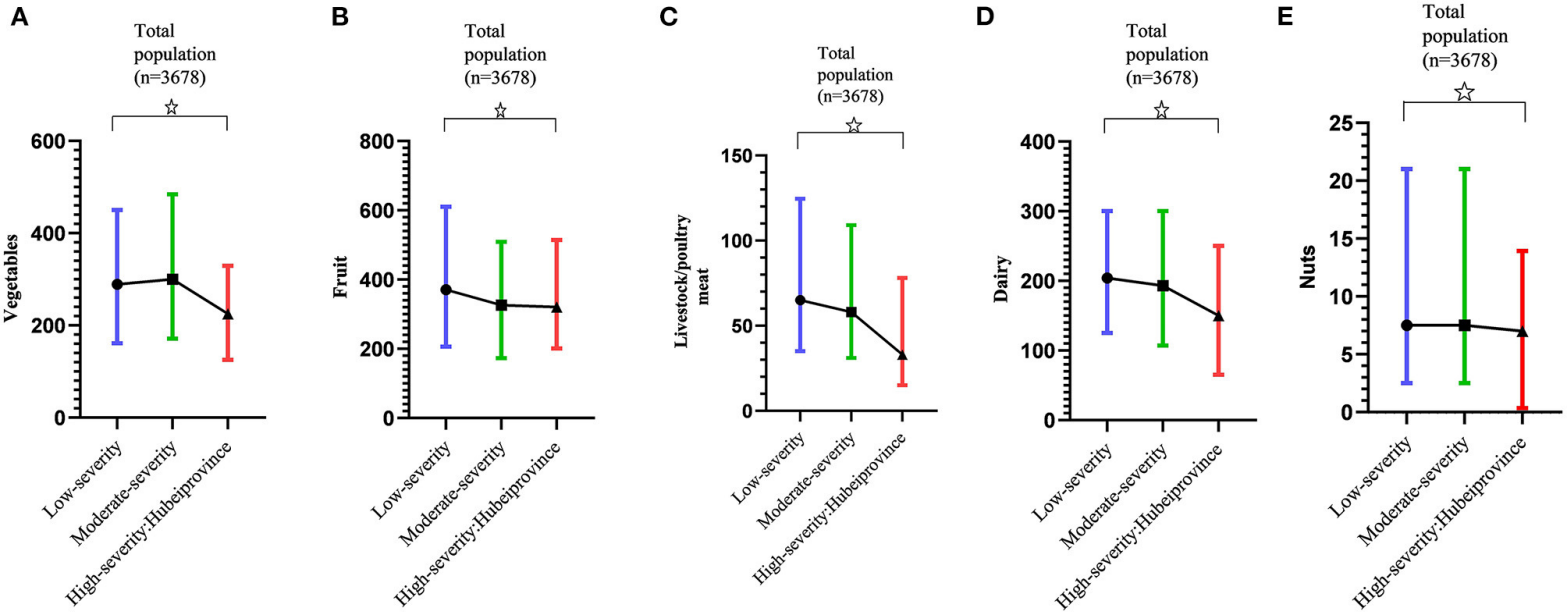
Variation of daily food intake according to COVID-19 pandemic severity. **(A)** Variation of daily intake of vegetables according to COVID-19 pandemic severity. **(B)** Variation of daily intake of fruit according to COVID-19 pandemic severity. **(C)** Variation of daily intake of livestock/poultry meat according to COVID-19 pandemic severity. **(D)** Variation of daily intake of dairy according to COVID-19 pandemic severity. **(E)** Variation of daily intake of nuts according to COVID-19 pandemic severity. Daily intake of vegetables, fruit, livestock/poultry meat, dairy and nuts were presented as median (interquartile range, IQR). ^✰^Linear trends were statistically significant (*p* < 0.05).

Variation in food intake among pregnant women according to regional COVID-19 pandemic severity is shown in Figure 3, revealing that the median daily intake of vegetables, fruit, livestock/poultry meat, dairy, and nuts decreased as pandemic severity increased (*p* < 0.05). When calculating the energy-adjusted intakes of foods, the same trends were seen in daily intake of vegetables, fruit, livestock/poultry meat, dairy, and nuts and pandemic severity (*p* < 0.05) (Supplementary Table 4). Of particular concern are areas with high pandemic severity (Hubei Province), whereas daily intake of vegetables, livestock/poultry meat, and dairy were notably lower than recommended (*p* < 0.05). No significant differences in daily intake of cereals/potatoes, eggs, and fish/shrimp were found across different severity levels (*p* > 0.05). Irrespective of pandemic severity, the median daily intake of cereals/potatoes exceeded the recommended ranges, and the daily intake of eggs and fish/shrimp was below recommended ranges (*p* < 0.05).

### Diet Quality According to COVID-19 Pandemic Severity

**Figure 4:**
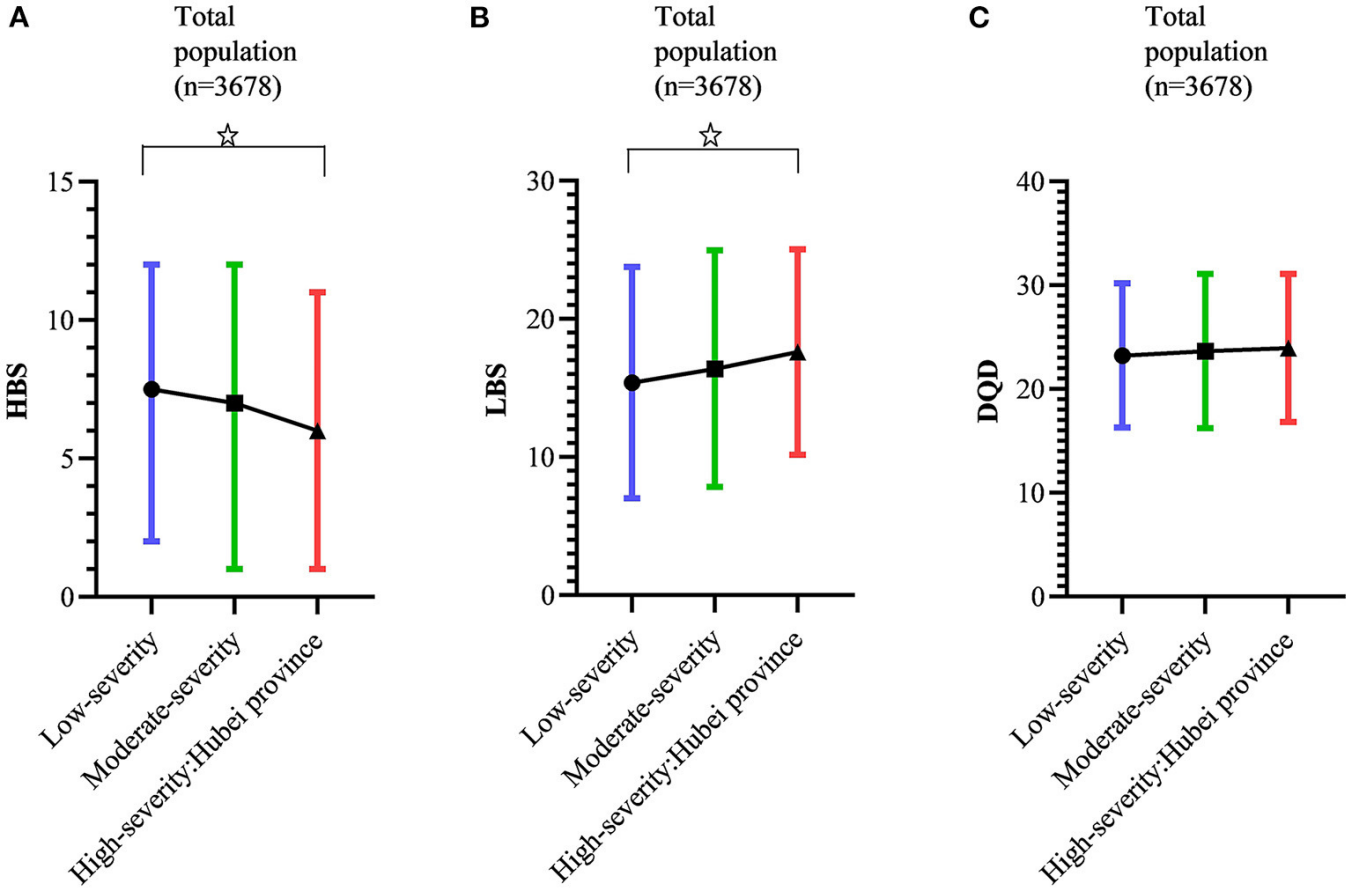
Variation of diet quality (Diet Balance Index for Pregnancy, DBI-P) according to COVID-19 pandemic severity. **(A)** Variation of high bound score (HBS) according to COVID-19 pandemic severity. **(B)** Variation of low bound score (LBS) according to COVID-19 pandemic severity. **(C)** Variation of diet quality distance (DQD) according to COVID-19 pandemic severity. HBS were presented as median (interquartile range, IQR). LBS and DQD were presented as mean (standard deviation, SD). ^✰^Linear trends were statistically significant (*p* < 0.05).

Figures 2, 4 show that in regions of low, moderate, and high pandemic severity, median HBS was 7.5, 7.0, and 6.0, respectively, suggesting a low degree of excessive intake, with decreasing HBS (lower excessive consumption) according to increased pandemic severity (*p* = 0.047). Similarly, the mean LBS was 15.4, 16.4, and 17.6, respectively, showing a low degree of insufficient consumption, with increasing LBS (greater insufficient consumption) as pandemic severity increased (*p* = 0.046). The mean DQD was 23.2, 23.7, and 23.9, respectively, signifying an overall moderate diet imbalance, but no significant DQD differences were found across pandemic severities (*p* > 0.05).

### Associations of Diet Quality With Pandemic Severity, Demographic Factors, and Health-Related Behaviors

The multivariate ordinal logistic regression analysis of HBS grade with COVID-19 pandemic severity, demographic factors, and health-related behaviors, revealed that HBS could be affected by pandemic severity. Specifically, a lower risk of excessive consumption was observed under moderate (OR = 0.687, 95% CI: 0.506–0.934, *p* = 0.017) and high (OR = 0.537, 95% CI: 0.315–0.916, *p* = 0.023) pandemic severity than under low severity conditions. Other potential factors influencing excessive dietary consumption are detailed in Table 3.

**Table 3:** Associations of diet quality (HBS) with COCID-19 pandemic severity, demographic factors, and health-related behaviors.

	**HBS (*****n*** **=** **3,678)**					
	**β coefficient (SE)**	**95% CI^**a**^**	**Wald *χ2***	***P*-value**	**OR**	**95% CI^**b**^**
**COVID-19 pandemic severity**						
High-severity^c^	−0.622 (0.273)	−1.156 to −0.088	5.206	0.023	0.537	0.315–0.916
Moderate-severity^c^	−0.375 (0.156)	−0.681 to −0.068	5.743	0.017	0.687	0.506–0.934
**Region**
North^d^	−0.314 (0.073)	−0.458 to −0.170	18.269	<0.001	0.731	0.633–0.844
**Educational level**
College degree or above^e^	0.077 (0.141)	−0.199 to 0.353	0.300	0.584	1.080	0.820–1.423
Senior school or technical secondary school^e^	0.385 (0.163)	0.066–0.703	5.598	0.018	1.470	1.068–2.020
**Hyperthyroidism in pregnancy**
No^f^	0.316 (0.176)	−0.029 to 0.661	3.213	0.073	1.372	0.971–1.937
Yes^f^	0.737 (0.280)	0.187–1.286	6.900	0.009	2.090	1.206–3.618
**Physical activity (MET-hours/week)**	0.002 (0.000)	0.001–0.003	13.870	<0.001	1.002	1.001–1.003
**Frequency of intake of calcium supplements**
None^g^	0.259 (0.229)	−0.189 to 0.707	1.289	0.256	1.296	0.828–2.028
1 time per day^g^	0.391 (0.227)	−0.054 to 0.836	2.968	0.085	1.478	0.947–2.307
4–5 times per week^g^	0.532 (0.259)	0.024–1.040	4.212	0.040	1.702	1.024–2.829
2–3 times per week^g^	0.059 (0.263)	−0.456 to 0.574	0.051	0.822	1.061	0.634–1.775

*HBS, high bound score; COVID-19, coronavirus disease 2019; SE, standard error; CI confidence interval; OR, odds ratio; MET, metabolic equivalent*.

a *95% CI for β coefficient*.

b *95% CI for OR*.

c *Compared to low-severity*.

d *Compared to south*.

e *Compared to less than senior school*.

f *Compared to not knowing*.

g *Compared to 1–2 times per month*.

*HBS was divided into three levels as dependent variables: no problem and almost no problem (control): 0–4; low and moderate level problem: 5–12; high level problem: 13–20. Adjusted for age, region, pre-pregnancy BMI, educational level, monthly income, assisted reproductive technology, gestational anemia, hyperthyroidism, and gestational diabetes mellitus, physical activity, the number of visits to hospital, the number of ways to acquire self-care and parenting knowledge, frequency of use of household fetal heart monitor, and frequency of intake of nutritional supplements (folic acid, and calcium). The multivariate ordinal logistic regression was performed for HBS. Statistically significant association (p < 0.05)*.

The dummy variable settings and the multiple factors influencing LBS and DQD are detailed in Table 4. Opposite associations were found between moderate and high pandemic severity and insufficient consumption, with the degree of insufficient consumption significantly higher in moderate (β = 1.517, 95% CI: 0.168–2.866, *p* = 0.028) and high (β = 3.020, 95% CI: 0.687–5.354, *p* = 0.011) pandemic severity than in low pandemic severity. The same relationships existed between moderate (β = 1.065, 95% CI: 0.186–1.943, *p* = 0.018), high (β = 2.945, 95% CI: 1.429–4.461, *p* < 0.001) pandemic severity and insufficient consumption when the energy-adjusted LBS was the dependent variable (Supplementary Table 5).

**Table 4:** Associations of diet quality (LBS, DQD) with COVID-19 pandemic severity, demographic factors, and health-related behaviors.

	**LBS (*****n*** **=** **3,678)**			**DQD (*****n*** **=** **3,678)**		
	**β coefficient (SE)**	**95% CI**	***P*-value**	**β coefficient (SE)**	**95% CI**	***p*-value**
**COVID-19 pandemic severity**						
High-severity^a^	3.020 (1.190)	0.687–5.354	0.011	–	–	–
Moderate-severity^a^	1.517 (0.688)	0.168–2.866	0.028	–	–	–
**Region**						
South^b^	−1.018 (0.318)	−1.640 to −0.395	0.001	–	–	–
**Educational level**						
College degree or above^c^	−3.388 (0.609)	−4.582 to −2.194	<0.001	−3.444 (0.521)	−4.467 to −2.422	<0.001
Senior school or technical secondary school^c^	−2.130 (0.711)	−3.524 to −0.735	0.003	−1.206 (0.616)	−2.413 to 0.001	0.050
**Monthly income (RMB)**						
>10,000^d^	−1.879 (0.519)	−2.896 to −0.863	<0.001	−1.830 (0.447)	−2.707 to −0.954	<0.001
5,000–10,000^d^	−1.035 (0.326)	−1.674 to −0.397	0.001	−1.281 (0.281)	−1.832 to −0.730	<0.001
**Assisted reproductive technology**						
Yes^e^	−1.272 (0.502)	−2.257 to −0.287	0.011	−1.338 (0.401)	−2.125 to −0.552	0.001
**Anemia in pregnancy**						
Not knowing^e^	1.468 (0.646)	0.201–2.735	0.023	1.090 (0.558)	−0.004 to 2.183	0.051
**Hyperthyroidism in pregnancy**						
Yes^e^	–	–	–	1.706 (0.861)	0.017–3.395	0.048
Not knowing^e^	2.273 (0.734)	0.833–3.713	0.002	1.807 (0.635)	0.562–3.053	0.004
**Diabetes mellitus in pregnancy**						
Yes^e^	–	–	–	−0.768 (0.370)	−1.494 to −0.042	0.038
**Physical activity (MET-hours/week)**	−0.007 (0.002)	−0.011 to −0.003	0.001	–	–	–
**The number of visits to hospital**						
0^f^	−1.103 (0.477)	−2.037 to −0.168	0.021	−0.826 (0.364)	−1.539 to −0.112	0.023
1–2^f^	−0.775 (0.329)	−1.420 to −0.129	0.019	–	–	–
**The number of ways to acquire self-care and parenting knowledge**						
3^g^	1.155 (0.305)	0.558–1.753	<0.001	1.217 (0.263)	0.701–1.733	<0.001
**Frequency of use of household fetal heart monitor**						
≥1 times per day^h^	−1.019 (0.570)	−2.136 to 0.098	0.074	−1.067 (0.488)	−2.024 to −0.110	0.029
4–5 times per week^h^	−2.383 (0.815)	−3.982 to −0.784	0.003	−1.750 (0.702)	−3.126 to −0.374	0.013
2–3 times per week^h^	−0.947 (0.572)	−2.068 to 0.174	0.098	−1.511 (0.492)	−2.477 to −0.545	0.002
1–2 times per month^h^	−0.921 (0.468)	−1.838 to −0.003	0.049	–	–	–
**Frequency of intake of folic acid supplements**						
4–5 times per week^i^	−2.640 (0.827)	−4.261 to −1.019	0.001	−1.229 (0.711)	−2.623 to 0.164	0.084
**Frequency of intake of calcium supplements**						
1 time per day^i^	−1.449 (0.298)	−2.034 to −0.864	<0.001	−1.076 (0.266)	−1.598 to −0.553	<0.001
2–3 times per week^i^	–	–	–	−1.200 (0.568)	−2.313 to −0.086	0.035
***R* ^2^**		0.060			0.075

*LBS, low bound score; DQD, diet quality distance; COVID-19, coronavirus disease 2019; SE, standard error; CI, confidence interval; MET, metabolic equivalent*.

a *Compared to low-severity*.

b *Compared to north*.

c *Compared to less than senior school*.

d *Compared to <5,000*.

e *Compared to no*.

f *Compared to ≥3*.

g *Compared to >3*.

h *Compared to none*.

i *Compared to 1–2 times per month*.

*Adjusted for age, region, pre-pregnancy BMI, educational level, monthly income, assisted reproductive technology, gestational anemia, hyperthyroidism, and gestational diabetes mellitus, physical activity, the number of visits to hospital, the number of ways to acquire self-care and parenting knowledge, frequency of use of household fetal heart monitor, and frequency of intake of nutritional supplements (folic acid, and calcium). –indicates the variable has been excluded from the model. The multivariate linear regression was performed for LBS and DQD. Statistically significant association (p < 0.05)*.

## Discussion

Despite the growing number of studies that have highlighted the potential effect of COVID-19 and related control measures on the dietary habits of the general population, little or no attention has been paid to evaluating the impact on the diets of pregnant women and it remains unclear whether the quantity and quality of food consumed by pregnant women varied according to COVID-19 pandemic severity. Detailed and differentiated dietary guidance for special groups is important both in the context of COVID-19 and for preparing for future potential pandemics. We used a national multi-center cross-sectional study to evaluate food intake and diet quality among pregnant women and assessed variations according to COVID-19 pandemic severity and related control measures. This research also included an examination of associations between diet quality and pandemic severity, demographic factors, and health-related behaviors.

### Overall Diet Quality of Pregnant Women During the COVID-19 Pandemic

The overall diet quality of study participants was moderately imbalanced, and 64.1% had moderate-to-high dietary imbalance. A study of the diets of pregnant women in Chengdu, China, conducted in 2013 and 2014 (27), found that 52.7% of participants had moderate to severe dietary imbalance, while a study in Shanghai in 2012 (28) indicated that 59.6% of the general population had moderate to high dietary imbalance. Compared to these pre-pandemic studies, a higher percentage of pregnant women had moderate to high dietary imbalance during the COVID-19 pandemic. Our findings are aligned with preliminary results showing that detrimental effect of COVID-19 pandemic and associated containment measures on diet quality (29). The consensus in the literature is that the effects of COVID-19 and related interventions on food and diet outcomes have been through their impacts on the food accessibility and availability, prices, income and associated purchasing power (30–32).

### Food Intake and Diet Quality According to COVID-19 Pandemic Severity

An imbalance in food consumption results from a combination of insufficient consumption excessive and consumption. The linear trend tests found that there was greater insufficient food consumption as pandemic severity increased, and this result was robust after adjustment for a number of demographic factors and health-related behaviors. Under more severe pandemic conditions, pregnant women consumed less quality food, including vegetables, fruit, livestock/poultry meat, dairy and nuts. The production and distribution of perishable and more nutritious foods are often more prone to disruptions during a crisis (33). The more serious the pandemic, the greater the impact on the many links in the food production chain, including livestock breeding, slaughtering, processing, transportation, distribution and storage (34). This may also explain this study's observation that meat consumption in Hubei Province was lower than recommended, while in other regions it remained adequate. The study also revealed that vegetables and dairy consumption in Hubei Province was lower than recommended. Our partial results are similar to a systematic review indicating that the great recession of 2008 caused a decrease in consumptions of foods such as fruit and vegetable (35). Two studies (36, 37) conducted in the Navajo nation found that access to and availability of foods are particularly important and determine food consumption. It is possible that during the COVID-19 pandemic, there was reduced consumption of foods with short shelf lives related to the number of shopping opportunities and locations. In several countries, the COVID-19 pandemic has disrupted access to food, led to food shortages and increased food prices (38, 39). As models have shown, during a pandemic, individuals reduce their consumption of animal protein, fruits, and vegetables due to the increased cost and availability (40). Moreover, the COVID-19 pandemic and the measures to contain the spread of virus exerted a profound deleterious effect on the employment, income and associated purchasing power, resulting in a decrease consumption of relatively expensive foods considered to be essential to a healthy diet include meat, vegetables and dairy (41). As one study in Vermont with 3,219 respondents found that there was nearly a one-third increase (32.3%) in household food insecurity (defined as limited or uncertain access to adequate amount of affordable and nutritious foods due to lack of economic resources) since COVID-19 (42). Long-term lack of access to nutritious foods has been found closely related to physical and mental health (43, 44).

Fruits and vegetables are rich in micronutrients that are essential to maintain physical health. These micronutrients include vitamins such as A, B, D, E, and C and trace elements, including selenium, zinc, iron, and magnesium. Vitamins and trace elements can support the integrity of epithelia, regulate the immune reactions, help sustain optimal function of the immune system and reduce the risk of infection such as COVID-19 (45–50). In addition to micronutrients, fruits, vegetables and nuts also contain high contents of antioxidant phytochemicals, mainly including polyphenols and carotenoids (51). Polyphenols and carotenoids play a crucial role in regulating immune response and protecting against diseases. For example, flavonoids, an important polyphenol substance, can regulate immune-related signaling molecules mRNA expression and facilitate growth and immune (52). Carotenoids can help the body produce immunoglobulin (Ig) and cytokine, and promote lymphocyte cytotoxic activity, which is crucial for resisting viruses, bacteria, fungi and parasites (53). Additionally, livestock/poultry meat, dairy, and nuts contain proteins, mainly made up of amino acids. Amino acids play an integral role in the activation T and B cells and the production of antibodies (54). Therefore, inadequate intakes of vegetables, fruit, livestock/poultry meat, dairy, and nuts can impair the structure and functions of immunity system and increase the risk of infections. We hereby recommend that in more severe pandemic situations, ensuring an increased supply of vegetables, fruit, and livestock/poultry meat, dairy, and nuts is very important.

Our study revealed that, although there was less excessive food consumption as pandemic severity increased, 60% of participants still experienced an excessive intake of cereals/potatoes. This was contrary to pre-pandemic results that more than 50% of the pregnant women showed the insufficient intake of cereals/potatoes (27). Firstly, the economic shutdowns, layoffs, and firm exits caused by the COVID-19 pandemic may result in an increase in the consumption of cheaper sources of calories like cereals/potatoes (41). Another reason for an increase in consumption of cereals/potatoes might be that they could be bought or stored in bulk. Furthermore, cereals/potatoes are rich in carbohydrates, which can alleviate stress due to serotonin production (55). In the face of COVID-19, pregnant women are exposed to enormous pressure, because they are disproportionately more likely to get infected and develop severe illness, require intensive care, and die than their non-pregnant counterparts (56). Elevated pandemic-related stress and anxiety may influence dietary habits like consuming more comfort foods high in carbohydrates to cope with stress. An article published in The Lancet (57) argued that both too many and too few carbohydrates in the diet can reduce life expectancy, and the optimal intake to maintain longer life expectancy is a dietary proportion of between 50 and 55%. Therefore, pregnant women are advised to follow these guidelines, and to consume carbohydrates in moderation.

### Associations of Diet Quality With Demographic Factors

Our study identified demographic characteristics affecting diet quality. Women in the north of the country suffered a higher degree of food deficiency than women in the south, while women in the south were more likely than women in the north to have excessive intake. This is due to differences in climate, culture, production, and lifestyle between the north and the south of China. In addition, women who had attained senior school, technical secondary school, or higher-level education experienced lower levels of deficiency and imbalance than women with an educational attainment below senior school. Women with senior school or technical secondary school attainment were at greater risk of excessive intake than women with college degree or above attainment. The study also found that diet tended to become more adequate and balanced with increased monthly income. Similar results have been found in other groups that the unprecedented COVID-19 pandemic and the associated economic and social responses are exerting greater influence on low-income, food-insecure households that already struggle to meet basic needs (58). Individuals with low incomes and/or limited formal education may have less flexibility in their jobs to allow them to earn income while staying home, or may be at higher risk of losing their jobs completely, thereby decreasing (or eliminating) incomes and reducing their diet quality (58). Consequently, people of low socioeconomic status experienced more severe effects of COVID-19 disasters in diet.

We found that women using assisted reproductive technology were far less likely to have insufficient and imbalanced diets compared to women with unassisted pregnancies. A relationship between pregnancy complications and diet quality also emerged. Women with hyperthyroidism were found to have more excessive diets. Excessive thyroid hormone secretion promotes metabolic activity, leading to patients often feeling hungry and having increased appetite. Women with GDM were also found to have a less imbalanced diet than women did not have GDM. This is a common and serious medical complication of pregnancy (59), and patients must pay strict attention to their diets. Physical activity was found to have a double effect on diet; increasing levels of physical activity improved dietary insufficiency, while dietary excess became severe. This result does not fully support the conclusion that individuals with higher levels of physical activity are more likely to follow healthy dietary guidelines (60). However, it supports the finding that those with higher levels of physical activity consume more food (61).

### Associations of Diet Quality With Health-Related Behaviors

In particular, we examined the associations between diet quality and health-related behaviors during the COVID-19 pandemic. Participants who visited hospitals at least three times had the lowest diet quality, compared to those who visited hospitals less than three times or had not been to hospital at all during the pandemic. Pregnant women who had more than three pathways to acquiring self-care and parenting information were associated with greater improvements to the quality of their diet than women with three or fewer pathways. Women who did not use a household fetal heart monitor had poorer dietary quality than those who used a household fetal heart monitor at different frequencies. Participants who supplemented their diets with folic acid and calcium 1–2 times per month had the most inadequate and imbalanced diets, compared to women who supplemented with folic acid 4–5 times per week and calcium once per day. These findings suggest the manifestation of the clustering of health behaviors (62).

## Conclusion

This nationwide multi-center cross-sectional study provides the first evidence that under more severe COVID-19 pandemic conditions, pregnant women consumed less quality food, characterized by reduced consumption of vegetables, fruit, livestock/poultry meat, dairy and nuts, while the quality of the foods that pregnant women consumed in excess tended to improve, but the overconsumption of cereals/potatoes was a problem. Also, the present study identified the potential demographic factors and health-related behaviors associated with diet quality during the COVID-19 pandemic. To further analyze the impact of the COVID-19 epidemic on the pregnant women's diet and the health of their offspring, the studies of dietary assessments throughout pregnancy during COVID quarantine and their associations with childbirth outcomes should be conducted.

## Limitations

The present study has some limitations. Due to the cross-sectional design of this study, the findings cannot isolate the specific mechanism or causal ordering of the effects. Also, the limitation to the study is the uncertainty of self-reporting food consumption due to its potential for social desirability bias and recall bias. Measurement error of self-reported demographic factors and health-related behaviors including pre-pregnancy BMI and physical activity is also inevitable. Finally, we did not compare pre-COVID-19 pandemic and during-COVID-19 pandemic food consumption, income, and so on. Future research could be more rigorous by observing participants and comparing data before and after COVID-19.

## Data Availability Statement

The datasets presented in this article are not readily available because of privacy and ethical restrictions. Requests to access the datasets should be directed to YZ, zhuyn3@mail.sysu.edu.cn.

## Ethics Statement

The studies involving human participants were reviewed and approved by the Ethics Committee of the School of Public Health, Sun Yat-Sen University (No. 017 [2020]). The patients/participants provided their written informed consent to participate in this study. Written informed consent was obtained from the individual(s) for the publication of any potentially identifiable images or data included in this article.

## Author Contributions

YZhu and ZW organized the research work. YZhu and HC conceived the study. HL interpreted the data, conducted an in-depth analysis, and wrote the manuscript. YC, HQ, YM, XB, YZha, LW, CL, JW, HW, and YJ were responsible for data gathering. YZhu was responsible for project administration. All authors contributed to the article and approved the submitted version.

## Funding

The study was funded by the Guangdong Provincial Natural Science Foundation (2021A1515010439, 2021A1515010411); the Sanming Project of Medicine in Shenzhen (SZSM201803061); the National Key R&D Program of China (2018YFC1002900); the Youth Cultivation Project of Sun Yat-sen University, China (17ykpy24); and the National Natural Science Foundation of China (81771606, 81571452).

## Conflict of Interest

The authors declare that the research was conducted in the absence of any commercial or financial relationships that could be construed as a potential conflict of interest.

## Publisher's Note

All claims expressed in this article are solely those of the authors and do not necessarily represent those of their affiliated organizations, or those of the publisher, the editors and the reviewers. Any product that may be evaluated in this article, or claim that may be made by its manufacturer, is not guaranteed or endorsed by the publisher.
