# Supplementary material for: Food Intake and Diet Quality of Pregnant Women in China During the COVID-19 Pandemic: A National Cross-Sectional Study
Source: Front Nutr. 2022 Apr 5;9:853565. doi: 10.3389/fnut.2022.853565 (PMC9037147; doi:10.3389/fnut.2022.853565)
Supplement: Supplementary file 1 [file Data_Sheet_1.doc]

| **Food items** | **First trimester** |  | **Second trimester** |  | **Third trimester** |
| --- | --- | --- | --- | --- | --- |
| Cereals/potatoes | 275–300 |  | 275–325 |  | 300–350 |
| Vegetables | 300–500 |  | 300–500 |  | 300–500 |
| Fruit | 200–350 |  | 200–400 |  | 200–400 |
| [Livestock/poultry meat](https://www.so.com/link?m=aoEhuYbeycZfGWuFNOOKPNQyp4s3ReLQG9ddJTvSX/0nB8bD3+OaNc8Rs0cxG9ziIgMIAo7lBOnhNMtuqAPFb1Ar+8X5jAgZ3jUqtJa3JEl2pyJ2p9gpzBY7IyiOOLbCXEqUgy4JMqkk3ahg4q9yFrqhBs/3i4OJYkBS8rZrFV73nIVXxsN7sXSuWzdK2eHVRLrHY6Dxj3RE=) | 40–65 |  | 50–75 |  | 75–100 |
| Fish/shrimp | 40–65 |  | 50–75 |  | 75–100 |
| Eggs | 45–60 |  | 45–60 |  | 45–60 |
| Nuts | 10 |  | 10 |  | 10 |
| Dairy | 300 |  | 300–500 |  | 300–500 |

**Supplemental Table 1.** Recommended food intake for Chinese pregnant women in different trimesters (g/d).

*Source: recommendation from the Chinese Balanced Dietary Pagoda for pregnant women.*

**Supplemental Table 2. Scoring method of Diet Balance Index for Pregnancy (DBI-P).**

| **Components** | **Score range** | **Subgroups** | **Score range** | **Scoring method** | | |
| --- | --- | --- | --- | --- | --- | --- |
|  |  |  |  | **First trimester** | **Second trimester** | **Third trimester** |
| Cereals and potatoes | (–12)–(12) | Cereals/potatoes | (–12)–(12) | 0 g=–12, (275–300) g=0,  >575 g=12  score increased 1 with intake amount increased 25 g | 0 g=–12, (275–325) g=0,  >600 g=12  score increased 1 with intake amount increased 25 g | ≤25 g=–12, (300–350) g=0,  >625 g=12  score increased 1 with intake amount increased 25 g |
| Vegetables  and fruit | (–12)–(0) | Vegetables  Fruit | (–6)–(0)  (–6)–(0) | 0 g=–6, ≥400 g=0  score increased 1 with intake amount increased 80 g | 0 g=–6, ≥400 g=0  score increased 1 with intake amount increased 80 g | 0 g=–6, ≥400 g=0  score increased 1 with intake amount increased 80 g |
|  |  |  |  | 0 g=–6, ≥275 g=0  score increased 1 with intake amount increased 55 g | 0 g=–6, ≥300 g=0  score increased 1 with intake amount increased 60 g | 0 g=–6, ≥300 g=0  score increased 1 with intake amount increased 60 g |
| Animal food | (–12)–(8) | [Livestock/poultry](https://www.so.com/link?m=aoEhuYbeycZfGWuFNOOKPNQyp4s3ReLQG9ddJTvSX/0nB8bD3+OaNc8Rs0cxG9ziIgMIAo7lBOnhNMtuqAPFb1Ar+8X5jAgZ3jUqtJa3JEl2pyJ2p9gpzBY7IyiOOLbCXEqUgy4JMqkk3ahg4q9yFrqhBs/3i4OJYkBS8rZrFV73nIVXxsN7sXSuWzdK2eHVRLrHY6Dxj3RE=)  [meat](https://www.so.com/link?m=aoEhuYbeycZfGWuFNOOKPNQyp4s3ReLQG9ddJTvSX/0nB8bD3+OaNc8Rs0cxG9ziIgMIAo7lBOnhNMtuqAPFb1Ar+8X5jAgZ3jUqtJa3JEl2pyJ2p9gpzBY7IyiOOLbCXEqUgy4JMqkk3ahg4q9yFrqhBs/3i4OJYkBS8rZrFV73nIVXxsN7sXSuWzdK2eHVRLrHY6Dxj3RE=)  Fish/shrimp  Eggs | (–4)–(4)  (–4)–(0)  (–4)–(4) | 0 g=–4, (42–70) g=0,  >112 g=4  score increased 1 with intake amount increased 14 g | 0 g=–4, (54–72) g=0,  >126 g=4  score increased 1 with intake amount increased 18 g | 0 g=–4, (75–100) g=0,  >175 g=4  score increased 1 with intake amount increased 25 g |
|  |  |  |  | 0 g=–4, ≥51 g=0  score increased 1 with intake amount increased 17 g | 0 g=–4, ≥63 g=0  score increased 1 with intake amount increased 21 g | 0 g=–4, ≥87 g=0  score increased 1 with intake amount increased 29 g |
|  |  |  |  | 0 g=–4, (45–60) g=0,  >105 g=4  score increased 1 with intake amount increased 15 g | 0 g=–4, (45–60) g=0,  >105 g=4  score increased 1 with intake amount increased 15 g | 0 g=–4, (45–60) g=0,  >105 g=4  score increased 1 with intake amount increased 15 g |
| Dairy and nuts | (–9)–(0) | Nuts  Dairy | (–3)–(0)  (–6)–(0) | 0 g=–3, ≥10 g=0  score increased 1 with intake amount increased 5 g | 0 g=–3, ≥10 g=0  score increased 1 with intake amount increased 5 g | 0 g=–3, ≥10 g=0  score increased 1 with intake amount increased 5 g |
|  |  |  |  | 0 g=–6, ≥300 g=0  score increased 1 with intake amount increased 60 g | 0 g=–6, ≥400 g=0  score increased 1 with intake amount increased 80 g | 0 g=–6, ≥400 g=0  score increased 1 with intake amount increased 80 g |
| Dietary variety | (–12)–(0) | Dietary variety | (–12)–(0) | Score is 0 when intake amount of each food group is more than 25 g, otherwise score is –1. | | |

**Supplemental Table 3.** Distribution of food intake and diet quality (DBI) for three trimesters during the COVID-19 pandemic.

|  | **First**  **trimester** | ***P* value**^†^ | **Second**  **trimester** | ***P* value**^§^ | **Third**  **trimester** | ***P* value**^‡^ | **Total**  **Population** | ***P* value**^*^ |
| --- | --- | --- | --- | --- | --- | --- | --- | --- |
|  | **(n=391)** |  | **(n=1078)** |  | **(n=2209)** |  | **(n=3678)** |  |
| Cereals/potatoes |  | <0.001 |  | 0.208 |  | 0.001 |  | 0.001 |
| Inadequate | 102 (26.1) |  | 229 (21.2) |  | 530 (24.0) |  | 861 (23.4) |  |
| Appropriate | 12 (3.1) |  | 94 (8.7) |  | 191 (8.6) |  | 297 (8.1) |  |
| Excessive | 277 (70.8) |  | 755 (70.0) |  | 1488 (67.4) |  | 2520 (68.5) |  |
| Vegetables |  | 0.626 |  | 0.003 |  | 0.131 |  | 0.007 |
| Inadequate | 254 (65.0) |  | 715 (66.3) |  | 1346 (60.9) |  | 2315 (62.9) |  |
| Appropriate | 137 (35.0) |  | 363 (33.7) |  | 863 (39.1) |  | 1363 (37.1) |  |
| Fruit intake |  | 0.001 |  | 0.904 |  | <0.001 |  | 0.002 |
| Inadequate | 141 (36.1) |  | 490 (45.5) |  | 1009 (45.7) |  | 1640 (44.6) |  |
| Appropriate | 250 (63.9) |  | 588 (54.5) |  | 1200 (54.3) |  | 2038 (55.4) |  |
| Livestock/poultry meat |  | <0.001 |  | <0.001 |  | <0.001 |  | <0.001 |
| Inadequate | 155 (39.6) |  | 488 (45.3) |  | 1262 (57.1) |  | 1905 (51.8) |  |
| Appropriate | 92 (23.5) |  | 147 (13.6) |  | 293 (13.3) |  | 532 (14.5) |  |
| Excessive | 144 (36.8) |  | 443 (41.1) |  | 654 (29.6) |  | 1241 (33.7) |  |
| Fish/shrimp |  | 0.001 |  | 0.005 |  | <0.001 |  | <0.001 |
| Inadequate | 319 (81.6) |  | 951 (88.2) |  | 2017 (91.3) |  | 3287 (89.4) |  |
| Appropriate | 72 (18.4) |  | 127 (11.8) |  | 192 (8.7) |  | 391 (10.6) |  |
| Eggs |  | 0.788 |  | 0.485 |  | 0.477 |  | 0.636 |
| Inadequate | 269 (68.8) |  | 723 (67.1) |  | 1452 (65.7) |  | 2444 (66.4) |  |
| Appropriate | 32 (8.2) |  | 98 (9.1) |  | 189 (8.6) |  | 319 (8.7) |  |
| Excessive | 90 (23.0) |  | 257 (23.8) |  | 568 (25.7) |  | 915 (24.9) |  |
| Nuts |  | 0.583 |  | 0.068 |  | 0.519 |  | 0.184 |
| Inadequate | 208 (53.2) |  | 556 (51.6) |  | 1214 (55.0) |  | 1978 (53.8) |  |
| Appropriate | 183 (46.8) |  | 522 (48.4) |  | 995 (45.0) |  | 1700 (46.2) |  |
| Dairy |  | 0.014 |  | 0.018 |  | 0.352 |  | 0.021 |
| Inadequate | 331 (84.7) |  | 963 (89.3) |  | 1909 (86.4) |  | 3203 (87.1) |  |
| Appropriate | 60 (15.3) |  | 115 (10.7) |  | 300 (13.6) |  | 475 (12.9) |  |
| Dietary variety score |  | 0.381 |  | 0.001 |  | 0.051 |  | <0.001 |
| 0 | 27 (6.9) |  | 74 (6.9) |  | 152 (6.9) |  | 253 (6.9) |  |
| –1 to –5 | 296 (75.7) |  | 848 (78.7) |  | 1835 (83.1) |  | 2979 (81.0) |  |
| – 6 to –10 | 68 (17.4) |  | 156 (14.5) |  | 222 (10.0) |  | 446 (12.1) |  |
| HBS |  | 0.649 |  | <0.001 |  | 0.072 |  | 0.001 |
| No problem | 71 (18.2) |  | 183 (17.0) |  | 473 (21.4) |  | 727 (19.8) |  |
| Almost no problem | 85 (21.7) |  | 233 (21.6) |  | 439 (19.9) |  | 757 (20.6) |  |
| Low level problem | 51 (13.0) |  | 174 (16.1) |  | 346 (15.7) |  | 571 (15.5) |  |
| Moderate level problem | 97 (24.8) |  | 247 (22.9) |  | 571 (25.8) |  | 915 (24.9) |  |
| High level problem | 87 (22.3) |  | 241 (22.4) |  | 380 (17.2) |  | 708 (19.2) |  |
| LBS |  | 0.012 |  | 0.001 |  | <0.001 |  | <0.001 |
| No problem | 4 (1.0) |  | 4 (0.4) |  | 7 (0.3) |  | 15 (0.4) |  |
| Almost no problem | 77 (19.7) |  | 202 (18.7) |  | 325 (14.7) |  | 604 (16.4) |  |
| Low level problem | 183 (46.8) |  | 599 (55.6) |  | 1340 (60.7) |  | 2122 (57.7) |  |
| Moderate level problem | 97 (24.8) |  | 200 (18.6) |  | 438 (19.8) |  | 735 (20.0) |  |
| High level problem | 30 (7.7) |  | 73 (6.8) |  | 99 (4.5) |  | 202 (5.5) |  |
| DQD |  | 0.154 |  | 0.003 |  | <0.001 |  | <0.001 |
| Almost no problem | 6 (1.5) |  | 7 (0.6) |  | 19 (0.9) |  | 32 (0.9) |  |
| Low level problem | 115 (29.4) |  | 360 (33.4) |  | 815 (36.9) |  | 1290 (35.1) |  |
| Moderate level problem | 207 (52.9) |  | 564 (52.3) |  | 1163 (52.6) |  | 1934 (52.6) |  |
| High level problem | 63 (16.1) |  | 147 (13.6) |  | 212 (9.6) |  | 422 (11.5) |  |

*Abbreviations: DBI, diet balance index; COVID-19, coronavirus disease 2019; HBS, high bound score; LBS, low bound score;*

*DQD, diet quality distance.*

*Statistically significant difference between ^†^ First vs. second trimester; ^§^ Second vs. third trimester; ^‡^ First vs. third trimester; ^*^ First vs.*

*second vs. third trimester* *(p < 0.05).*

*The data are presented as frequency (percentages). Percentages may not total 100 because of rounding.*

**Supplemental Table 4.** Variation of average energy-adjusted daily food intake and diet quality (DBI-P) according to COVID-19 pandemic severity.

|  | **Low-severity** | **Moderate-severity** | **High-severity** | ***P* value** |
| --- | --- | --- | --- | --- |
|  | **(n=158)** | **(n=3425)** | **(n=95)** |  |
| Cereals/potatoes | 476.5 ± 115.3 | 468.0 ± 105.0 | 479.0 ± 90.0 | 0.062 |
| Vegetables | 291.5 ± 289.0 | 314.0 ± 321.0 | 225.0 ± 236.0 | 0.049 |
| Fruit intake | 404.0 ± 298.3 | 375.0 ± 274.5 | 370.0 ± 266.0 | 0.037 |
| Livestock/poultry meat | 71.0 ± 95.0 | 65.0 ± 89.0 | 33.0 ± 65.0 | <0.001 |
| Fish/shrimp | 17.0 ± 26.0 | 20.0 ± 30.0 | 20.0 ± 27.0 | 0.889 |
| Eggs | 43.0 ± 38.5 | 42.0 ± 41.0 | 44.0 ± 30.0 | 0.881 |
| Nuts | 9.6 ± 16.5 | 9.9 ± 14.9 | 7.3 ± 12.64 | 0.015 |
| Dairy | 212.0 ± 162.3 | 204.0 ± 182.0 | 150.0 ± 212.0 | 0.042 |
| HBS | 7.0 ± 3.3 | 7.0 ± 4.0 | 7.0 ± 3.0 | 0.888 |
| LBS | 12.5 ± 8.0 | 14.0 ± 8.0 | 15.0 ± 8.0 | 0.001 |

*Abbreviations: DBI-P, diet balance index for pregnancy; COVID-19, coronavirus disease 2019; HBS, high bound score; LBS, low bound score; IQR, interquartile range.*

*The data are presented as median ± IQR.*

*Linear* *trend tests were performed to assess variation of average energy-adjusted daily food intake, HBS and LBS according to COVID-19 pandemic severity.*

*Statistically significant (p < 0.05).*

**Supplemental Table 5. Associations of energy-adjusted diet quality (LBS) with COVID-19 pandemic severity, demographic factors, and health-related behaviors.**

|  | **Energy-adjusted LBS (n=3678)** | | |
| --- | --- | --- | --- |
|  | **β coefficient (SE)** | **95% CI** | ***P* value** |
| COVID-19 pandemic severity |  |  |  |
| High-severity^a^ | 2.945 (0.773) | 1.429 to 4.461 | <0.001 |
| Moderate-severity^a^ | 1.065 (0.448) | 0.186 to 1.943 | 0.018 |
| Region |  |  |  |
| South^b^ | –0.684 (0.207) | –1.090 to –0.278 | 0.001 |
| Educational level |  |  |  |
| College degree or above^c^ | –2.397 (0.396) | –3.173 to –1.621 | <0.001 |
| Senior school or technical secondary school^c^ | –1.430 (0.462) | –2.337 to –0.524 | 0.002 |
| Monthly income (RMB) |  |  |  |
| >10000^d^ | –1.428 (0.338) | –2.090 to –0.766 | <0.001 |
| 5000–10000^d^ | –0.964 (0.212) | –1.380 to –0.548 | <0.001 |
| Assisted reproductive technology |  |  |  |
| Yes^e^ | –0.843 (0.327) | –1.484 to –0.201 | 0.010 |
| Anaemia in pregnancy |  |  |  |
| Not knowing^e^ | 0.998 (0.420) | 0.175 to 1.822 | 0.018 |
| Hyperthyroidism in pregnancy |  |  |  |
| Not knowing^e^ | 1.073 (0.478) | 0.135 to 2.011 | 0.025 |
| Physical activity (MET-hours/week) | –0.005 (0.001) | –0.008 to –0.003 | <0.001 |
| The number of ways to acquire self-care and parenting knowledge |  |  |  |
| 3^g^ | 0.930 (0.199) | 0.541 to 1.319 | <0.001 |
| Frequency of use of household fetal heart monitor |  |  |  |
| 4–5 times per week^h^ | –1.788 (0.530) | –2.827 to –0.749 | 0.001 |
| Frequency of intake of folic acid supplements |  |  |  |
| 4–5 times per week^i^ | –1.446 (0.539) | –2.503 to –0.389 | 0.007 |
| 2–3 times per week^i^ | –1.184 (0.566) | –2.294 to –0.074 | 0.037 |
| Frequency of intake of calcium supplements |  |  |  |
| 1 time per day^i^ | –1.007 (0.194) | –1.387 to –0.628 | <0.001 |
| R^2^ | 0.073 | | |

*Abbreviations: LBS, low bound score; COVID-19, coronavirus disease 2019; SE, standard error; CI, confidence interval; MET, metabolic equivalent.*

*^a^ Compared to low-severity; ^b^ Compared to north; ^c^ Compared to less than senior school; ^d^ Compared to <5000; ^e^ Compared to no; ^g^ Compared to >3; ^h^ Compared to none; ^i^ Compared to 1–2 times per month.*

*Adjusted for age, region, pre-pregnancy BMI, educational level, monthly income, assisted reproductive technology, gestational anemia, hyperthyroidism, and gestational diabetes mellitus, physical activity, the number of visits to hospital, the number of ways to acquire self-care and parenting knowledge, frequency of use of household fetal heart monitor, and frequency of intake of nutritional supplements (folic acid, and calcium).*

*The multivariate linear regression was performed for LBS. Statistically significant association (p < 0.05).*

**Supplemental Figure 1.** Average daily food intake and diet quality (Diet Balance Index, DBI) for three trimesters during the COVID-19 pandemic.

**(A)** The median daily intake of cereals/potatoes, vegetables, fruit and dairy (g/day) for three trimesters during the COVID-19 pandemic. **(B)** The median daily intake of livestock/poultry meat, fish/shrimp, eggs and nuts (g/day) for three trimesters during the COVID-19 pandemic. **(C)** The median of high bound score (HBS), the mean of low bound score (LBS) and diet quality distance (DQD) for three trimesters during the COVID-19 pandemic.. ^§^ *p* < 0.05 (second vs. third trimester). ^‡^ *p* < 0.05 (first vs. third trimester). ^*^ *p* < 0.05, ^d^ *p* < 0.001 (first vs. second vs. third trimester).
